# Diagnostically Oriented Experiments and Modelling of Switched Reluctance Motor Dynamic Eccentricity

**DOI:** 10.3390/s21113857

**Published:** 2021-06-03

**Authors:** Jakub Lorencki, Stanisław Radkowski, Szymon Gontarz

**Affiliations:** Institute of Vehicles and Construction Machinery Engineering, Faculty of Automotive and Construction Machinery Engineering, Warsaw University of Technology, Narbutta 84, 02-524 Warszawa, Poland; stanislaw.radkowski@pw.edu.pl (S.R.); szymon.gontarz@pw.edu.pl (S.G.)

**Keywords:** SRM, FEMM, eccentricity, diagnostics

## Abstract

The article compares the results of experimental and modelling research of switched reluctance motor at two different operational states: one proper and one with mechanical fault, i.e., with dynamic eccentricity of the rotor. The experiments were carried out on a test bench and then the results were compared with mathematical modelling of quasi-static and dynamic analysis of 2D geometry model. Finally, it was examined how the operation with dynamic eccentricity fault of the motor affected its main physical parameter—the phase current. The analysis was presented in the frequency domain using the Fast Fourier Transform (FFT); however, individual current waveforms in the time domain are also shown for comparison. Applying results of the research could increase reliability of the maintenance of SRM and enhance its application in vehicles for special purposes as well as its military and industrial applications.

## 1. Introduction—The Aim of the Study

**Figure 1:**
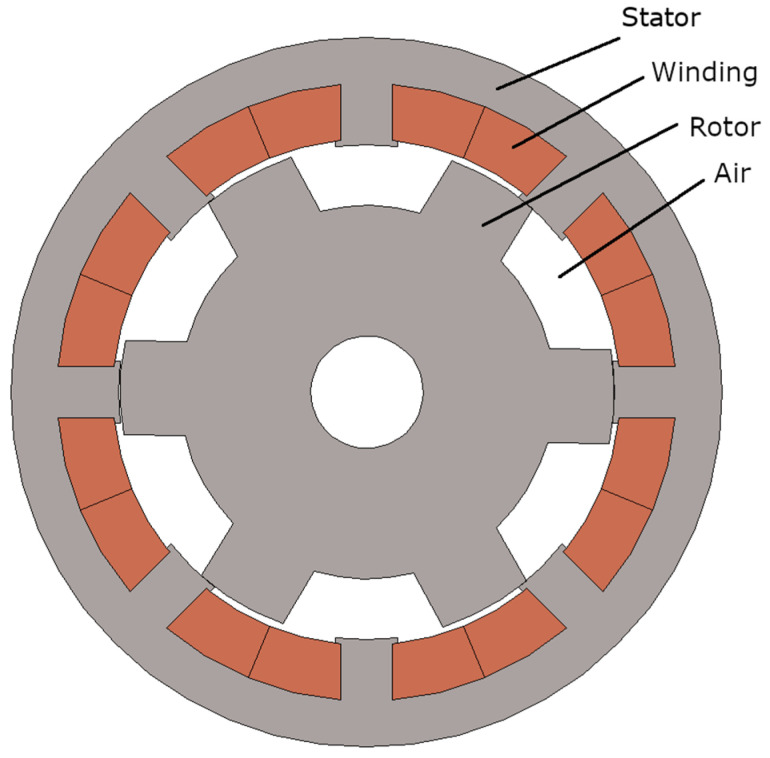
SRM Cross section with visible stator and rotor salient poles.

The switched reluctance motor (SRM) is a brushless motor made of iron (electrotechnical iron sheets) with electronic commutation—the power is supplied by transistor half-bridge. It is known for its simple construction and increased durability in comparison to another electrical motors. The SRM is able to reach very high rotational speed even above 10,000 rpm. For this reason, this motor is more popular in special applications, such as in the military, industry, transportation, etc. Nonetheless, it can also be applied in conventional electric and hybrid electric vehicles. Given the development of this type of vehicle in recent years due to environmental tightening, the usage of this motor might become even more widespread in the future [1]. Another factor that contributes to the mechanical reliability of the motor is the simplicity of its construction, characterized by salient poles on the stator and on the rotor and windings located only on the stator (Figure 1), which is helpful for the cooling. Nevertheless, the SRMs also have several disadvantages such as torque pulsation, tendency to vibrations and noise [2].

The SRM, as with any other electric motor, can also undergo every type of mechanical or electrical damage. The operation of the motor with such faults could result in accelerated damage to the components directly cooperating with the drive as well as lead to increased degradation and destruction of the motor itself. Therefore, a diagnostically oriented model analysis of such a situation, and above all an experiment that verifies the model, will allow for their proactive maintenance. Such an approach would increase the reliability of systems using SRMs and their popularity in the application to a variety of objects.

**Figure 2:**
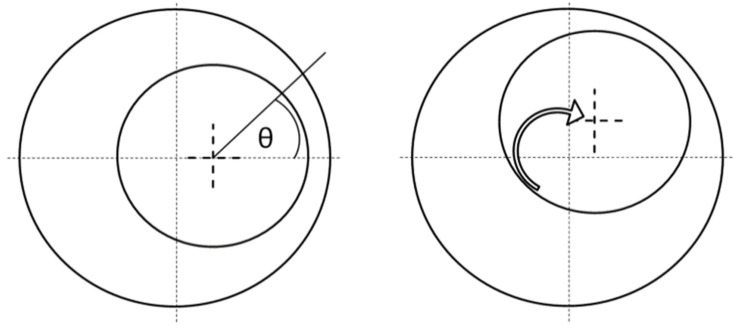
Depiction of static and dynamic eccentricity of the motor.

One of the mechanical failures found in electric motors is eccentricity and there are two types of it: static and dynamic (Figure 2). In static eccentricity, the centerline of the shaft is at constant offset from the center of the stator, thus non-uniform air-gap is also constant. In dynamic eccentricity, the offset between the shaft’s centerline and stator varies in time—changing the air-gap length in time.

The experiment was carried out for two cases of electric motor operation: one nominal and one with 80% degree of rotor dynamic eccentricity (i.e., occurring at 80% of total air-gap length). The research was conducted as an experiment on a test bench and then was verified by mathematical modeling using magnetostatic FEA method with the FEMM software resulting in dynamic variables obtained with the Simulink package. Since the characteristics of the reluctance motor operation are non-linear and during this type of research many undesirable phenomena occur (vibrations and other factors related to interference in the motor structure), verification of the model was necessary to answer the question whether the phenomena occurring after the failure come from the alteration in the motor’s operation or from external interference.

The current signal in the case of introduced rotor eccentricity contains information about the damage of the electric motor, which is diagnosed in the frequency domain by the Fast Fourier Transform (FFT). Based on such information, the diagnostician can predict the degradation of the electric motor in advance and prevent costly failure or unexpected downtime of some device.

Regarding the mechanical diagnostics of electric motors, the literature mainly covers the topic of induction motors, which are known to be the most widely used electric motor for industrial applications [3,4,5]. As for the reluctance motor, since it is not a popular motor, there is relatively little literature on the subject. These are only descriptions of various types of modeling and simulation; there are no experiments on the physical object regarding mechanical fault diagnostics. In [6], the authors simulate the SRM motor performance at time-varying conditions using Wigner–Ville distribution. In [7,8,9,10,11,12,13,14,15,16], the authors present different approaches for motor modelling either 2D or 3D with different physical phenomena. In [17], there is a fine introduction to SRM modeling with comparison to test bench analysis but without any diagnostic components. 

In [18], the authors relate to the electrical diagnosis of the electric motor power supply circuit, i.e., short circuit fault and open circuit in half bridge. 

Article [19] is only about modeling the fault of the rotor position sensor and stator windings. Admittedly, it is also diagnostics (in this case only modeling), but also based only on the electrical issue. 

The lack or small number of described experimental tests with such a defect is probably due to the fact that the construction of such a stand that allows the introduction of a given damage and modification in the structure of the motor construction is a problematic and dangerous matter.

The paper is divided into five chapters: introduction, presentation of test bench experiments, modelling analysis, its results and then conclusion. 

## 2. Test Bench Experiments 

### 2.1. Test Bench Construction

**Figure 3:**
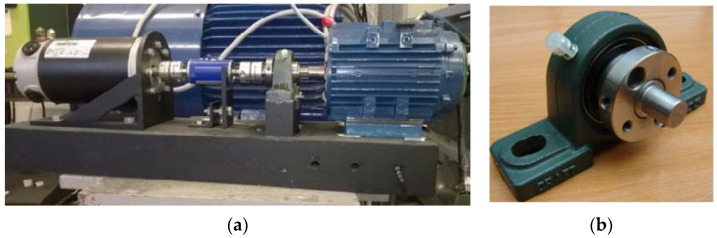
Test bench elements for eccentricity (**a**) Test bench with assembled switched reluctance motor on the right, also (from the left) DC motor, torquemeters, clutches and sleeve; (**b**) Sleeve with bearing support to cause eccentricity.

**Figure 4:**
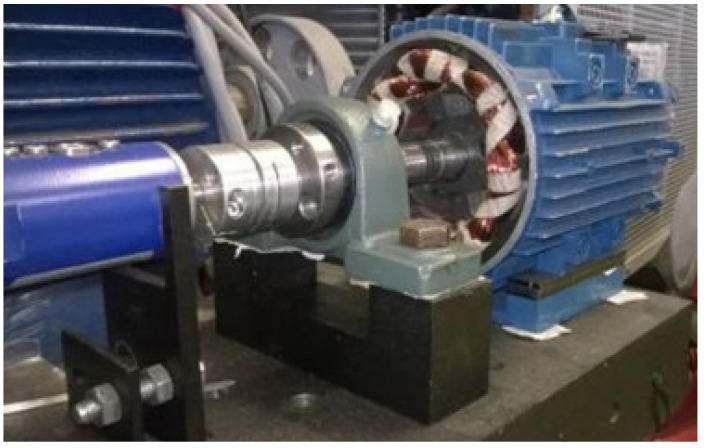
Switched reluctance motor mounted on a test bench with visible rotor poles.

The first stage of the task was an experiment on a test stand, where the reluctance motor was mechanically interfered by causing damage of dynamic eccentricity onto it. For this reason, the test bench was constructed (Figure 3a) consisting of the following elements: a reluctance motor, a DC electric motor that acted as a dynamometer, clutches, a torque meter and a special sleeve for setting the rotor eccentricity. In addition, equipment from National Instruments (Compact DAQ) for data collection and current clamps for non-invasive measurement of the phase current value were connected. A sleeve was designed to move the rotor axis away from the stator axis by an offset of 0.25 mm (Figure 3b) which was about 80% of its air-gap length. The stand made possible to test the electric motor in its entire range of operation, therefore it was necessary have a load with freely regulated torque applying the load to the tested motor. In Figure 4, the SRM is mounted on a test bench with dismantled housing with visible rotor poles. This operation was necessary to remove the bearing. The operation to conduct a dynamic eccentricity test on a physical motor is very difficult. This requires a mechanical intervention in the motor structure and a lot of precautions must be taken. 

The tested SRM with a nominal power of 550 W was manufactured by Huayang electric especially for this research (Table 1). Between the tested electric motor and the load, there was a torquemeter connected by two clutches. For the needs of the station, electronic systems have been designed that allow manual control of the operation of the tested and loaded electric motor. Appropriate systems also ensured the conditioning of the measurement signals and their adjustment to levels acceptable for National Instruments measurement cards.

The DC motor control system was implemented as a current source with adjustable efficiency. Both systems allowed for manual control of the stand and automatic control using appropriate analog signals, which allowed for some automation of measurements. A software in the LabView environment was created for the purposes of data registration and initial analysis.

### 2.2. Results from Experiment

**Figure 5:**
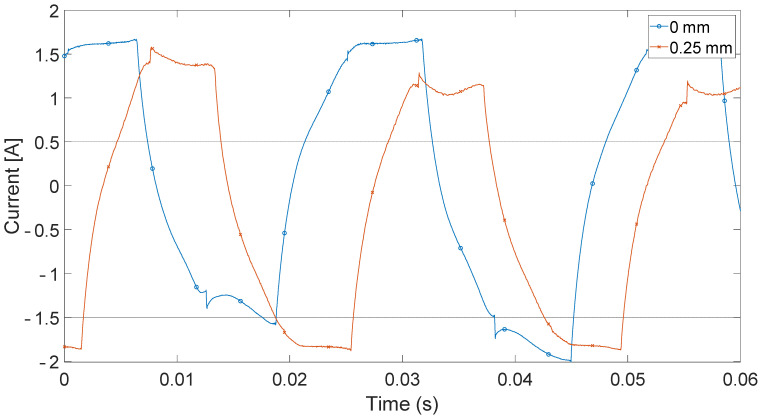
Phase current measured on test bench.

**Figure 6:**
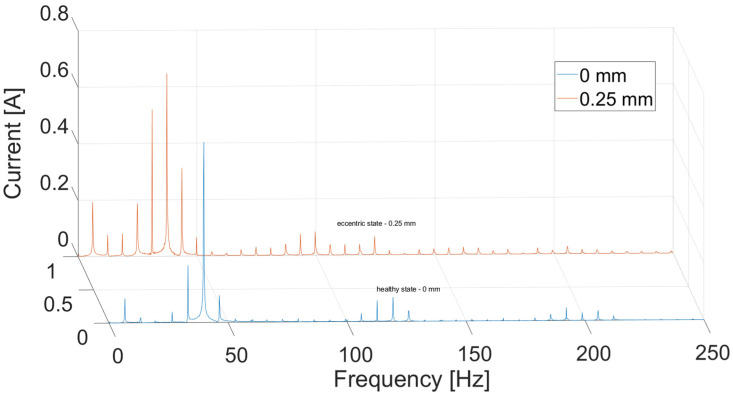
Phase current measured on test bench in frequency domain.

For the analysis, current signals in the frequency domain were compared using FFT. Only one time-domain waveform was given as an example for comparison (Figure 5). In Figure 6, there are results of the current signal spectrum after FFT analysis where there are clearly visible data peaks with the same distance of 6.66 Hz (i.e., 400 rpm) from each other for both cases of electric motor operations. The figure also shows that the sixth peak has the highest value, and it is surrounded by slightly smaller ones around it, especially in the case of faulty operation. This phenomenon occurs to a lesser extent for the third (120 Hz) and fifth (200 Hz) multiplication of the sixth peak. The geometry of the SRM rotor of six salient poles makes the sixth peak the highest (i.e., 6.66 × 6 = 39.96 Hz). This effect results from the air-gap size decrease and the nonlinearity related to the magnetic field occurring there. Amplitude and phase modulations visible here result from the construction characteristics of the motor (salient poles, nonlinearity of magnetic field) and to a small extent from the construction of the test stand and the noise associated with it. 

The question remains: which of these phenomena result strictly from the changed motor operation mode, and which may result from external reasons related to the stand itself and its imperfection. The answer to this may be the modeling of such a phenomenon, which will be presented in the next chapter. However, on the basis of these data, it is possible to try to determine the envelope in order to find the value of amplitude and phase modulation using the Hilbert transform, which will be done in further work [20,21,22,23].

## 3. SRM Modelling

### 3.1. Modelling Assumptions

**Figure 7:**
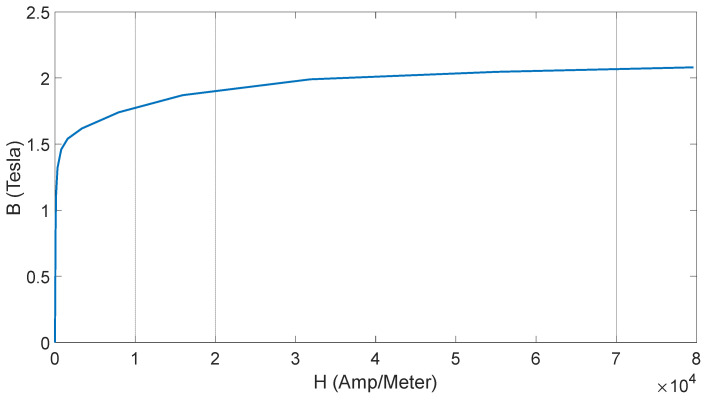
M-19 sheet magnetization graph.

In order to confirm the authenticity of the experiment results in theory, an electromagnetic analysis was carried out in the form of a magnetostatic and electrodynamic model. For generating an asymmetrically magnetic circuit in SRM, the number of stator and rotor poles must be different. Usually, the number of poles on the stator is greater than that on the rotor. In the cross section of such a motor shown in Figure 1, a shape of the rotor and stator can be observed with a ratio of stator to rotor poles equal to 8/6. Here, there are only three types of materials—iron (electrical sheet marked with symbol M-19 with the characteristics shown in Figure 7), copper (used in the windings) and air. 

**Figure 8:**
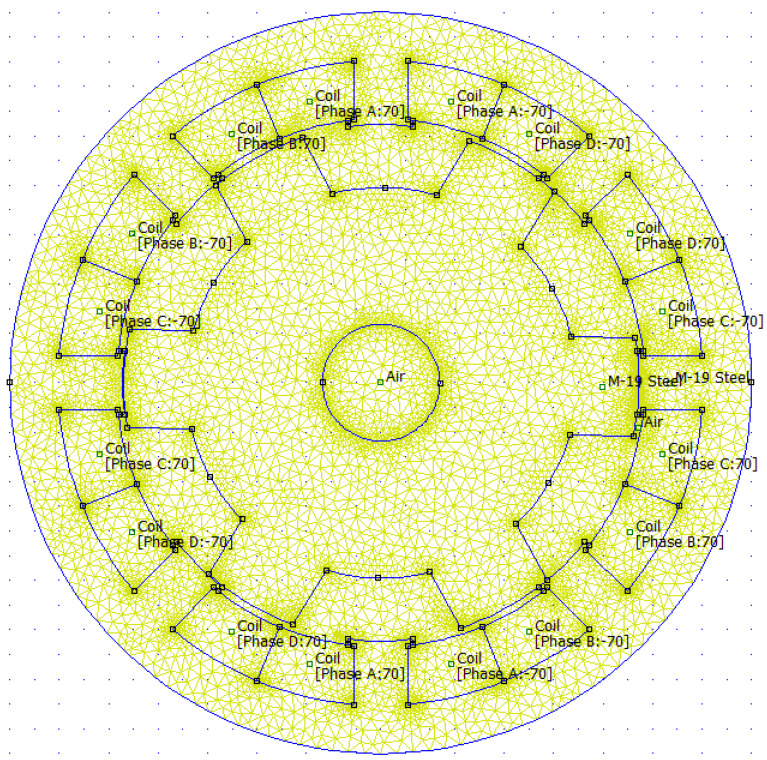
Mesh and material boundaries created in FEMM package for SRM.

**Figure 9:**
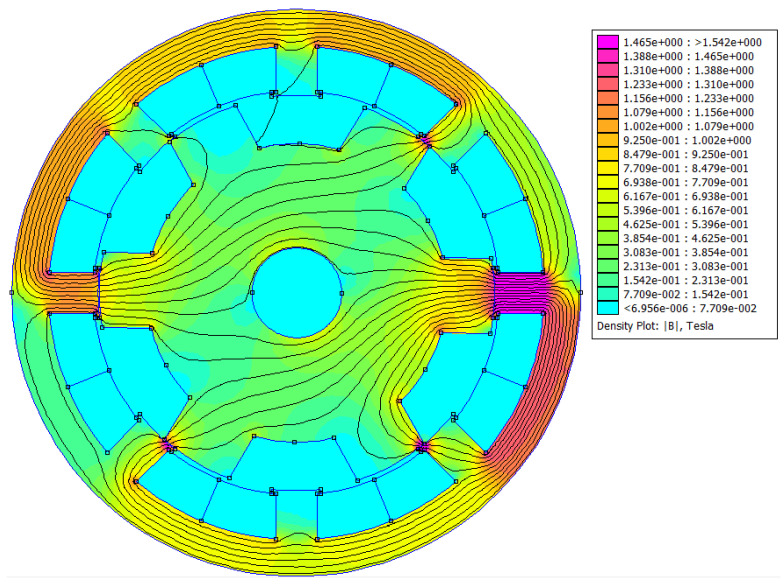
Density plots for SRM Motor.

The cross-section of SRM will be analyzed by means of finite element method analysis by the FEMM package (Finite Element Magnetic Method). The solver by means of generated mesh (Figure 8) uses magnetostatic analysis to calculate magnetic field strength lines and based on it, it is possible to obtain the value of flux densities ***B*** in each triangle (Figure 9). Knowing the magnitude of B and the surface of the motor, the software integrates these values in order to determine the magnetic flux magnitude. With given rotation angle and current in windings it is possible to calculate other physical quantities in motor, as ***k_e_***—quotient of magnetic flux and angle of rotation, inductance ***L*** (quotient of magnetic flux and current) and finally the electromagnetic torque ***T_e_***. 

The air-gap resulting from the design of the motor must still be designed as small as possible precisely because of the low loss of magnetic flux, and hence the inductance and current value. Dynamic eccentricity equal to 80% was chosen in order to obtain as high difference between each state as possible.

The analysis will be carried out for two cases: for the correct rotation of the rotor for movement along a changed path corresponding to the case of dynamic eccentricity (0.25 mm—80% of air-gap length. 

### 3.2. Parameters of the Examined Motor

Table 1 presents the parameters of the tested reluctance motor. These data show that this motor has a compact size, low nominal power and can be used to power household appliances, small vehicles, etc. The cross-section of this motor is shown in Figure 1. 

### 3.3. Physical Quantities in Modeling

In the following equations, the influence of currents from other phases on the flux associated with a given phase were ignored. This means that the flux associated with a given phase depends only on the current in that phase. In the case of concentrated windings wound on salient poles, such simplification is acceptable because the mutual inductances are about ten times smaller than the self-inductances.

The induced voltage in the inductive element modelling the *k*-th winding phase is given by the equation [16]:

(1) $$U_{k}\left(i_{k},\alpha \right)=\frac{d\psi_{k}\left(i_{k},\alpha \right)}{dt}$$

Taking into account the dependence of the flux associated with the *k*-th phase on *m* currents and the angular position of the rotor the following equation results:

(2) $$U_{k}\left(i_{k},\alpha \right)=k_{Ek}\left(i_{k},\alpha \right)\frac{d\alpha }{dt}+L_{k}\left(i_{k},\alpha \right)\frac{di_{k}}{dt}+Ri_{k}$$

The first component is called the rotation voltage because it depends on the angular velocity of the rotor $\frac{d\alpha }{dt}$. The second component is called the transformation voltage, because it depends on the variability in time of individual currents $\frac{di_{l}}{dt}$. The derivative of the magnetic flux associated with the *k*-th phase after the mechanical coordinate—the angular position is called the coefficient of induced rotation voltage.

(3) $$k_{Ek}\left(i,\alpha \right)=\frac{\partial \psi_{k}\left(i_{k},\alpha \right)}{\partial \alpha }$$

Partial derivatives of the flux after-currents, which, as with the flux, are also functions of the currents and the position of the rotor, define the dynamic inductances of the winding:

(4) $$L_{k}\left(i,\alpha \right)=\frac{\partial \psi_{k}\left(i_{k},\alpha \right)}{\partial i_{k}}$$

The electromagnetic torque acting on the moving part of the transducer is defined as a partial derivative of magnetic co-energy after mechanical coordinate. The mechanical coordinate is the angular position of the rotor, so the electromagnetic torque acting on the rotor is determined by the relationship [20]:

(5) $$T_{e}\left(i,\alpha \right)=\frac{\partial }{\partial \alpha }W_{m}^{\prime }\left(i_{k},\alpha \right)$$

where: $W_{m}^{\prime }$—magnetic co-energy accumulated in the motor volume:

(6) $$W_{m}^{\prime }\left(i_{k},\alpha \right)=\overset{m}{\underset{k=1}{\sum }}\overset{i_{k}}{\underset{0}{\int }}\psi_{k}\left(i_{k}^{\prime },\alpha \right)di_{k}^{\prime }$$

where: $i_{m}^{\prime }$—the currents in the phase winding given as the coordinates on which the flux depends, as opposed to specific current values *i*_1_, *i_2_*, …, *i_m_* [24,25,26].

## 4. Model Results

The magnetic flux was calculated directly by FEMM software; then, using the Equations (1)–(5) the following values were calculated using Simulink dynamic model: voltage coefficient ***k_E_***, inductance ***L***, co-energy ***W*** and torque ***T_e_***. The values were compared for dynamic eccentricity as well as for normal operation.

**Figure 10:**
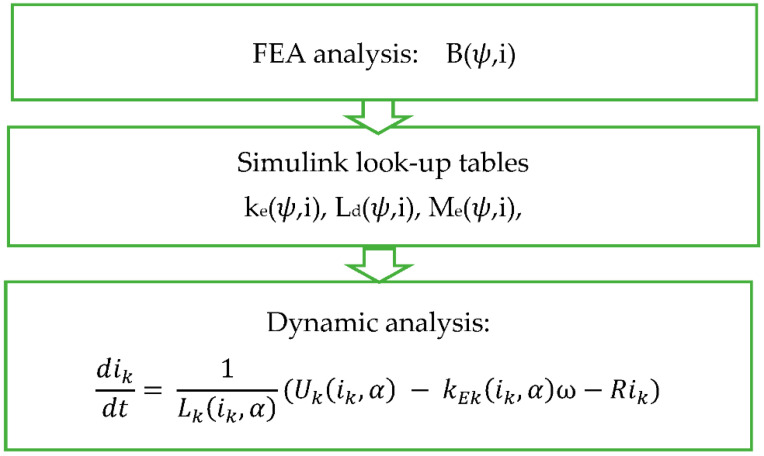
Scheme of SRM Modelling.

Figure 10 shows a short diagram of modelling procedure of the dynamic value of current over time. The current value was derived from the Equation (2). The quantities that cannot be calculated algebraically due to the nonlinearity of the motor (ψ,i) were determined using the finite element methods of the FEMM program.

**Figure 11:**
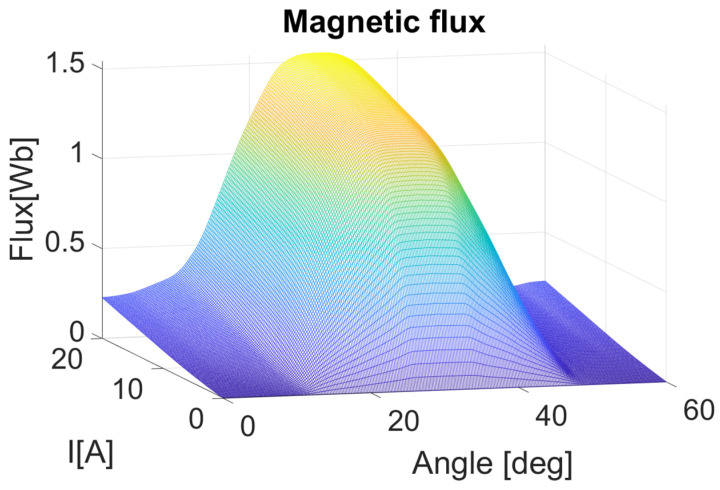
Flux mesh graph of one phase at different current and angle values.

As it was mentioned earlier, the magnetic flux values were calculated first directly by the FEMM package as a function of the rotation angle and as a function of the current value as shown in Figure 11. As the rotor pole approaches the stator, the flux value increases and reaches maximum value for an angle of 30 degrees, because there is a coil with a winding with such position of the rotor angle. After that point, the flux value drops with the same rate. In this three-dimensional dependence of the flux on the current and the angle of rotation, it can be seen that for a lower current, the dependence on the angle is linear and saturation occurs with the increase in current value. Other physical quantities were also determined in this way: both for the healthy condition and with the introduced eccentricity.

**Figure 12:**
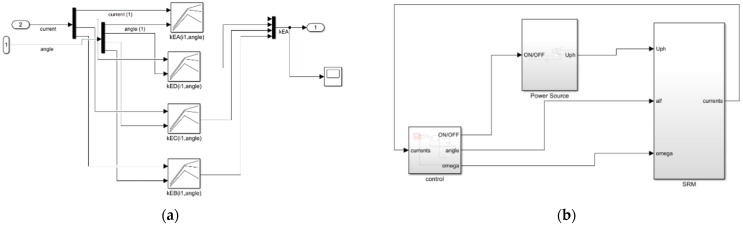
Dynamic SRM modeling in Simulink (**a**) Fragment of Simulink code responsible for loading look-up tables of *k_e_* values; (**b**) Simulink model overview of SRM.

Having the static values calculated they were used consequently in the lookup-tables of Simulink software. These values were: kE, LD and TE. Fragment of the Simulink program is shown in Figure 12a,b. These values were then used in voltage Equation (2) of SRM motor model. In that model the phase was firing up for 15 degrees.

**Figure 13:**
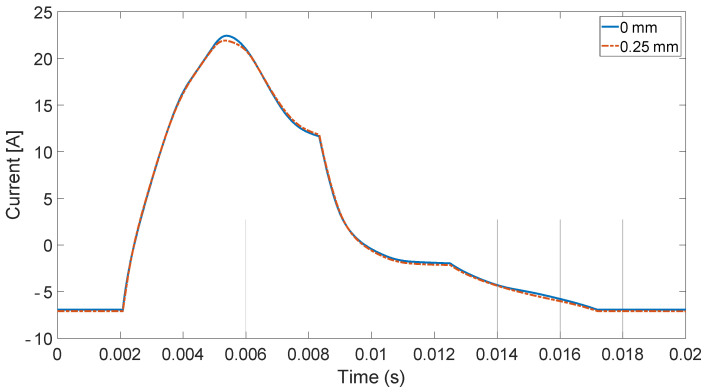
Phase current.

**Figure 14:**
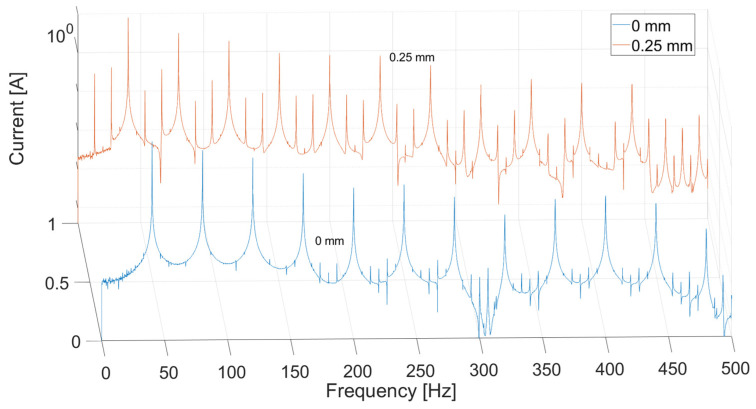
Phase current in frequency domain of constant voltage source in logarithmic scale.

**Figure 15:**
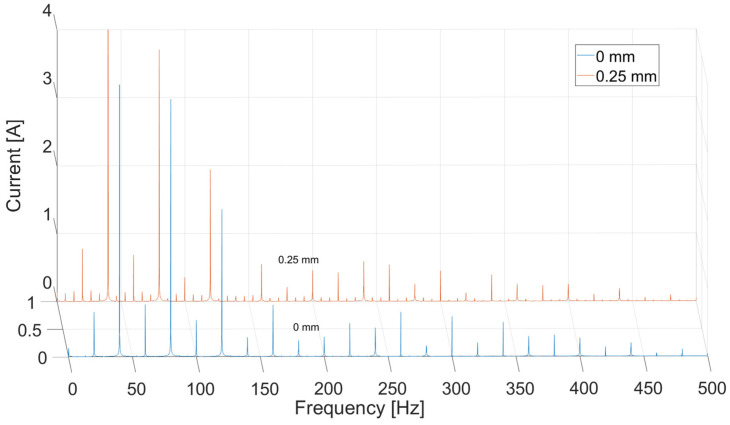
Phase current in frequency domain of sinusoidal source with 2 additional harmonics.

In Figure 13, there is a simulation result for the current in time domain for two types of operation. The differences between two experiments occur only for the falling slopes. The rising slopes are the same in both cases. Figure 14 and Figure 15 present a comparison of the magnitude of the current in the frequency domain using the Fast Fourier Transform (FFT) in two cases: for a DC voltage source and for sinusoidal voltage with introduced third and fifth harmonics. The first graph (DC voltage) is shown on a logarithmic scale in order to show details of the signal in greater detail. It shows the highest frequency peak around 40 Hz, due to the fact that it is six times the value of 6.66, which corresponds to a rotational speed of 400 Hz. In the state of dynamic eccentricity, there are two peaks between the large peaks, especially visible between the first and the sixth peak. The distance between them is approximately 13 Hz, i.e., twice the value of the rotational speed. At higher frequencies, even smaller data peaks occurring every one rotational speed value can be observed.

On the other hand, the clearest correlation between the experimental research and the model occurs at an absolute value of sinusoidal source with introduced harmonics (Figure 15). The figure shows the similarity in the occurrence of both harmonics equal to one rotational speed (6 Hz) and the occurrence of the greatest harmonic as the sixth (due to the fact that the motor has six poles on the rotor), along with the decreasing occurrence of successive harmonics in the form of vanishing sines.

## 5. Conclusions

The results have shown that the current signal can be a suitable physical quantity for the analysis of SRM mechanical fault of dynamic eccentricity, because there is a clear difference between the state of proper operation of the electric motor and the state with the introduced damage. These differences are visible both in the results of the experiment on the test bench and in mathematical modeling. Thanks to this, the obtained results can be used to develop remaining useful life (RUL) models [27], but of course also for early detection of the emerging eccentricity [28].

The comparison of the results between the theoretical and experimental analysis showed interesting correlation in frequency domain analysis of current signal. Unsurprisingly, the results on the test stand showed more interesting phenomena distinguishable in the signal and more differences between them. There are higher modulation values around the data peaks in the faulty state most prominent around the sixth peak.

Results from model-based data were similar to those from experiment, especially in the form of absolute value of sinusoidal input. It can be observed on the graphs that frequency peaks are at a distance equal to the rotational speed (6.66 Hz) and other signal features that occurred in the experimental test: the highest possible value at the frequency of the sixth multiplication of rotational speed and the presence of other harmonics in the signal. The analysis of the phenomenon occurs from the first to the fifth harmonic. Successive values increase and they are a feature of noise.

Certainly, the minor differences between experimental and modeling research are influenced by physical factors coming from the operation of the measuring station itself, such as vibrations of the entire system, loose joints and others. The signal can also be influenced by a power supply system or losses in electronic components, such as transistors or diodes. This work focuses on the analysis of amplitude modulation, while phase modulation studies can answer further questions. Such an analysis may indicate which of these phenomena result strictly from the design properties of the electric motor and its nominal magnetic parameters, and which results from the imperfection of the experimental stand.

The results of this work can be placed in an appropriate database in the future and used to build a model based on machine learning.

## Figures and Tables

**Table 1:** SRM Motor parameters.

Motor Parameter	Value
number of phases	4
stator poles	6
rotor poles	8
coil turns per winding	70
motor length	250 mm
stator outer diameter	117 mm
air-gap length	0.31 mm
rotor outer diameter	60.79 mm
shaft diameter	4.73 mm
stator inner diameter	62 mm
height of tooth	5.5 mm
width of tooth tip	3.5 mm
height of tooth foot	0.5 mm
width of tooth	3 mm
rotor inner diameter	12.73 mm
width of lobe	3 mm
height of lobe	2.51 mm
rated power	500 W
nominal speed	1500 rpm
supply voltage	315 V DC
